# Non-Invasive Detection of Fetal Vascular Endothelial Function in Gestational Diabetes Mellitus

**DOI:** 10.3389/fendo.2021.763683

**Published:** 2021-10-29

**Authors:** Yunyu Chen, Danping Huang, Jinrong Liu, Fangling Zeng, Guoyi Tang, Wenjia Lei, Haiyu Wang, Yanmin Jiang, Weihui Shentu, Hongying Wang

**Affiliations:** ^1^ Department of Medical Ultrasonics, Guangzhou Women and Children’s Medical Center, Guangzhou Medical University, Guangzhou, China; ^2^ Institute of Perinatal Nutrition, Guangzhou Women and Children’s Medical Center, Guangzhou Medical University, Guangzhou, China; ^3^ Institute of Obstetrics and Gynecology, Guangzhou Women and Children’s Medical Center, Guangzhou Medical University, Guangzhou, China

**Keywords:** gestational diabetes mellitus (GDM), echocardiography, aortic propagation velocity, fetus, endothelial dysfunction

## Abstract

**Objectives:**

Endothelial dysfunction in the fetuses of women with gestational diabetes mellitus (GDM) is associated with their subsequent cardiovascular events. Prenatal assessment of endothelial function in fetuses exposed to intrauterine hyperglycemic environment remains challenging. The aim of this study was to assess the fetal vascular endothelial function in GDM patients using color M-mode derived aortic propagation velocity (APV) and evaluate the correlation of APV with endothelial function biomarkers.

**Methods:**

This observational cross-sectional study included 31 gestational diabetic mothers and 30 healthy pregnant mothers from August 2019 to January 2020. Clinical data were compared between the groups. Fetal APV was measured using color M-mode echocardiography at late gestation. Concentrations of endothelial biomarkers including von Willebrand Factor (vWF), vascular endothelial-cadherin and endothelin-1 in umbilical cord serum were assessed. Measurements between diabetic group and controls were compared.

**Results:**

vWF was the only endothelial functional marker that differed between the two groups. Fetuses in the GDM group had significantly lower APV levels and higher vWF levels compared with the healthy controls (*P* < 0.05). There was a moderate but significant correlation between APV and vWF (r =−0.58, *P* < 0.001). There were no associations between APV and ventricular wall thickness or umbilical artery pulsatility index.

**Conclusions:**

Color M-mode propagation velocity of aorta is a non-invasive, practical method that correlates well with GDM and fetal endothelial function. This novel metric could contribute to recognizing early vascular functional alterations and hence represents a potential strategy for early risk factor surveillance and risk modification.

## Introduction

Gestational diabetes mellitus (GDM) is the most common complication during pregnancy (1) and has both short-term and long-term effects on the child after birth, such as an increased risk for metabolic syndrome, including cardiovascular disease (CVD) (2–4). The hyperglycemic environment is associated with fetal endothelial dysfunction, which is considered the first stage in the development of atherosclerosis and other CVD (5). Therefore, assessment of fetal endothelial function is critical to help identify individuals with vascular risks. Endothelial dysfunction can lead to impaired vasodilation, increased leukocyte adhesion to the vascular wall and increased platelet aggregation, and therefore an increased propensity to develop diabetes-associated CVD (6, 7). Previous experimental studies have reported an increased impaired human umbilical vein endothelial cells and elevated damaged cell markers in fetal cord blood at birth, which were produced under a stressful hyperglycemic condition (8–12). However, prenatal evaluation of the effect of GDM on fetal endothelial function remains difficult. Although flow-mediated vasodilation (FMD) is considered the gold standard method to determine vascular endothelial function (13), this technique is difficult to perform in a fetus because it requires the placement of a blood pressure cuff around the upper arm. Recent studies have shown that color M-mode-derived propagation velocity of the descending thoracic aorta (APV) correlates well with FMD in some CVDs, such as coronary artery disease, coronary slow flow and atherosclerosis in adults, which begin with endothelial injury and progress to clinically overt diseases (14–17). APV is thus a potential simple and non-invasive surrogate marker of endothelial dysfunction in adults. However, no studies have examined the association between APV measurements and endothelial dysfunction in the fetus.

We hypothesized that GDM would induce fetal endothelial dysfunction and APV measurements may help recognize early vascular alterations in fetuses exposed to GDM. Here we evaluated the validity of APV measurement in comparison with biomarkers of endothelial dysfunction assessed by the fetal cord blood and assessed the impact of hyperglycemia on fetal endothelial function. To the best of our knowledge, this is the first demonstration of fetal endothelial function using this non-invasive and simple method.

## Methods

### Study Population

This observational cross-sectional study of consecutive singleton pregnancies at 24–40 weeks of gestation was performed from August 2019 to January 2020 at Guangzhou Women and Children’s Medical Center. We recruited these patients at the time of a 75-g oral glucose tolerance test (OGTT); GDM was diagnosed at 24–28 weeks when one or more of the venous plasma glucose measurements met or exceeded the following thresholds after a 75-g OGTT: fasting blood glucose ≥ 5.1 mmol/L (92 mg/dl); 1 h plasma glucose level ≥ 10.0 mmol/L (180 mg/dl); or 2 h plasma glucose level ≥ 8.5 mmol/L (153 mg/dl), as recommended by the International Association of the Diabetes and Pregnancy Study Groups (18). Patients diagnosed with GDM received nutritional counseling including dietary and physical activity advice. Patients were requested to self-monitor their blood glucose levels at home and return to the nutrition doctor every two or three weeks. Our criteria for well controlled GDM were as follows: fasting blood glucose <5.0–5.5 mmol/L and postprandial blood glucose <6.7–7.1 mmol/L, according to the treatment targets recommended in the guidelines of the American Diabetes Association (19). When capillary blood glucose level met the criteria for one month, GDM was considered as well controlled. All consecutive women with singleton pregnancies with negative OGTT findings were also recruited during this period. Exclusion criteria for patient inclusion in the study were maternal CVD, pre-gestational diabetes, pre-existing hypertensive disorder, structural or chromosomal fetal anomalies, evidence of fetal infection, multiple gestations and maternal chronic disease other than diabetes mellitus. Women who were unable to deliver in our hospital, dropped out of the study halfway and fetuses with poor ultrasound image quality or without available fetal cord blood were excluded from the analysis.

### Ethical Approval

The study was approved by the ethics committee of the Guangzhou Women and Children’s Medical Center before patient recruitment (approval number # 017A01). Written informed consent to publish clinical details and images was obtained from all participants before the study. This study was performed according to the principles of the Declaration of Helsinki.

### Maternal Characteristics

The baseline variables, including maternal age, height, weight, smoking, and history of GDM and cesarean delivery, were collected at the time of the ultrasound examination. Additionally, fasting blood glucose, 1 h plasma glucose level and 2 h plasma glucose level in 75-g OGTT and glycated hemoglobin A1 (HbA1c) were recorded. Body mass index of all mothers was calculated.

### Fetal Echocardiographic Examination and Measurements

Gestational age was determined based on the beginning of the last menstrual period and identified by sonographic crown-rump length measurement in the first trimester. Routine prenatal screening was conducted to exclude intracardiac and extracardiac malformations. For women diagnosed with GDM, a comprehensive fetal echocardiography was conducted to detect congenital cardiac anomalies using a Philips ultrasound machine (iE33; Philips Medical Systems, Andover, MA, USA) with a C5-1 MHz abdominal transducer.

**Figure 1:**
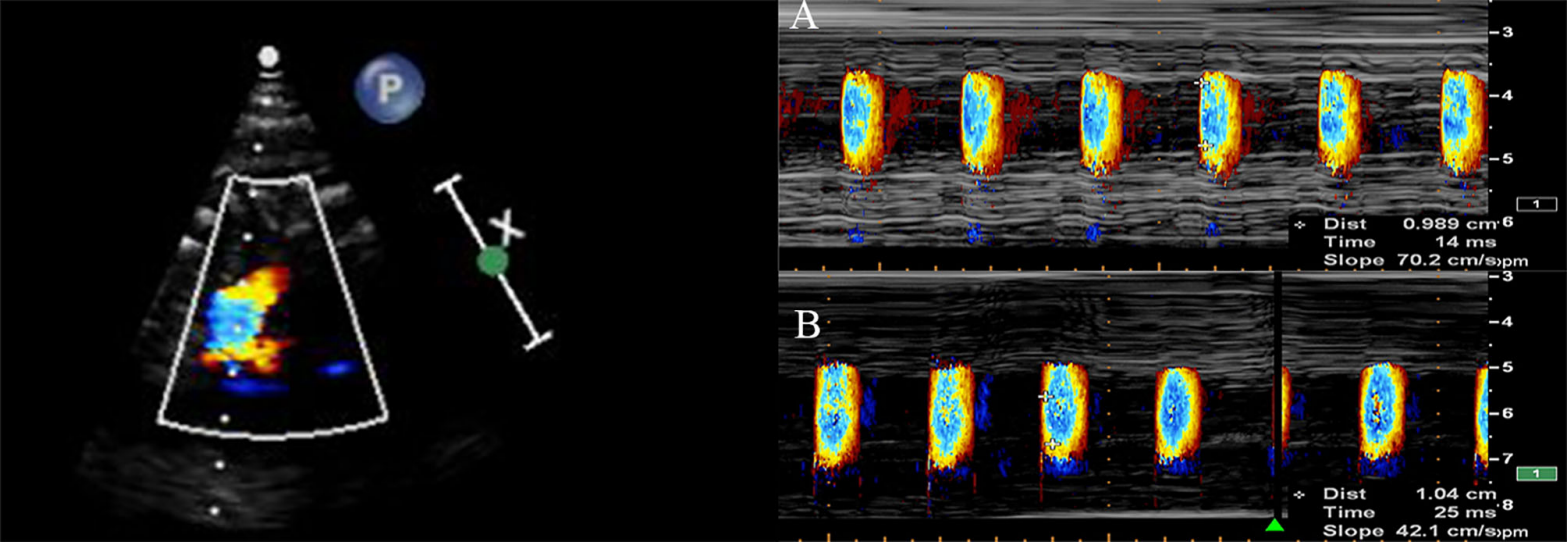
The aortic propagation velocity evaluated by color M-mode echocardiography display across the aorta arch from the sagittal view in a normal fetus **(A)** and in a fetus exposed to GDM **(B)**.

At 36–40 weeks, fetal vascular functional assessments were performed at the time of the routine ultrasound examination before delivery. Fetal color M-mode Doppler assessments were performed and recorded on the sagittal plane of the aorta arch. Because of the difficulty to position the cursor parallel to the main flow of the descending aorta in the intrauterine condition, we aimed for an aliasing velocity between 30 and 50 cm/s and adjusted to M-mode with a recorder sweep rate of 75 mm/s. A flamed shaped M-mode spatiotemporal velocity map was then shown (**Figure 1**). Visual assessment was made to ensure a clear delineation of the isovelocity slope and, if necessary, baseline shifting was performed to change the aliasing velocity until a clear delineation of the aliasing contour was displayed. APV was measured by tracing the velocity slope of the first aliasing contour corresponding to the time. Thus, APV refers to the velocity at which the flow is propagating down the artery. If the aliasing contour was not distinct enough because of fetal movements, such as body and breathing movements, we excluded the contour and continued until we obtained a clear aliasing contour. If the fetal heart was not properly visualized in the appropriate insonation angle because of a suboptimal fetal position, the mother was re-examined after walking for 20–30 min. Each sonographic image was measured by an experienced fetal echocardiographer (H.W.) who was blinded to the maternal characteristics and GDM status. To minimize intraobserver variability, measurements were repeated three times, and the average of the three measurements was used as the APV value. Intra- and interobserver reproducibilities of fetal APV were assessed by two operators. A random sample of 19 cases was selected for analysis. The first operator (Operator A) (H.W.) performed the assessments three times for the intraobserver test. A second operator (D.H.) performed an assessment blinded to the results of the first operator for interobserver variability. To assess intraobserver and interobserver variability, intraclass correlation coefficients (ICC) and 95% confidence interval (CI) were assessed. Ventricular wall thickness including thickness of the left ventricular lateral wall (LVLW), interventricular septum (IVS), and right ventricular free wall (RVFW) and the umbilical artery pulsatility index (UA-PI) were measured and recorded.

### Serum Collection and Biomarker Measurement

Venous umbilical cord blood was taken immediately from the placental side of the cord after the delivery and cord clamping. Immediately after blood collection, blood was centrifuged at 3000 rpm for 10 min (Thermo Fisher Scientific, USA), and the serum was separated into four 0.5 mL aliquots and frozen at −80°C (Thermo Fisher Scientific) until evaluation. Concentrations of vWF (Von Willebrand Factor), vascular endothelial-cadherin (VE-cadherin) and endothelin-1 (ET-1) in umbilical cord serum were measured using commercial ELISA kits (Cusabio, Wuhan, China) according to the manufacturer’s protocols. The absorbance was read at 450 nm with a microplate reader (BioTek, USA). The concentrations of vWF, VE-cadherin and ET-1 were calculated based on a standard curve.

### Statistical Analysis

Descriptive data are presented as mean (± standard deviation) for normally distributed variables and as median and interquartile range for skewed distributions. Data were analyzed using SPSS version 25.0 (SPSS Inc., Chicago, IL, USA). Maternal and fetal characteristics were compared between GDM and controls with the independent samples Student’s T test for normally distributed quantitative variables or the Mann–Whitney U test for variables without a normal distribution and the chi-squared test for categorical variables, respectively. Correlations between variables were assessed by Spearman correlation coefficients tests. All probability values were two-sided, and *P* < 0.05 was considered to indicate statistical significance.

## Results

The initial screening identified 165 consecutive participants who were eligible for the study. Among this group, 32 patients opted to not participate, 3 had poor ultrasound images or incomplete imaging and 2 showed hypertensive disorder; antepartum data was incomplete for 2 participants because of premature birth, and umbilical cord blood was not collected for 65 participants because of urgent delivery, short cord, coagulated blood, not delivering in our center. Finally, 61 patients were enrolled in the study, including 30 patients diagnosed with GDM and 31 patients as non-GDM controls. There was no maternal smoking in the overall patient group. Most patients with GDM had good glycemic control after lifestyle and diet intervention.

The clinical maternal and fetal characteristics for the GDM and control groups are summarized in **Table 1**. The maternal baseline characteristics were mostly similar between the two groups, except for glucose levels in OGTT test and HbA1c levels, which were higher in the GDM group. UA-PI, ventricular wall thickness including LVLW, IVS and RVFW, gestational age at delivery, and birth weight did not differ significantly between the groups. Patients in the GDM cohort had relatively higher levels of ET-1 and lower levels of VE-cadherin levels compared with controls; however, these differences did not reach statistical significance. Among the examined biomarkers (vWF, ET-1 and VE-cadherin), only vWF exhibited differences between the two groups, with significantly higher levels in the GDM group than in the controls. Fetuses born from patients with GDM had significantly lower APV values compared with controls (*P* < 0.001, **Table 2**). In addition, APV was significantly correlated with vWF (r=−0.58, *P* < 0.001) (**Table 3**). The Spearman correlation analysis also revealed a significant (*P* < 0.001) correlation between APV values and HbA1c as well as glucose level of 75-g OGTT. The ICC for Operator A was 0.961 (95% CI, 0.920–0.984), and the ICC between the two observers was 0.883 (95% CI, 0.726−0.953).

**Table 1:** Characteristics of the study population.

Characteristics	Controls (N = 31)	GDM (N = 30)	*P* value
Age (years)	29.84 ± 3.07	31.00 ± 2.92	0.14
BMI (kg/m2)	21.00 (17.97 – 29.69)	21.76 (17.80 – 27.58)	0.08
Fasting blood glucose (mmol/L)	4.29 (3.88 – 4.94)	4.78 (3.64 – 7.41)	**0.001**
1-h plasma glucose (mmol/L)	6.91 (3.92 – 9.80)	10.45 (3.32 – 14.62)	**0.001**
2-h plasma glucose (mmol/L)	6.46 ± 1.18	9.10 ± 1.73	**0.001**
HbA1c	5.20 (4.70 – 5.60)	5.50 (4.80 – 6.70)	**0.001**
History of cesarean delivery	5 (16.13%)	5 (16.67%)	1^*^
History of GDM	0	3 (10.00%)	0.23^*^
Multiparity	14 (45.16%)	19 (63.33%)	0.15
Gestational age at delivery (weeks)	39.30 (37.30 – 41.0)	39.30 (37.20 – 40.1)	0.68
Cesarean or forceps delivery	11 (35.48%)	10 (33.33%)	0.86
Neonate features			
Sex Male/Female	16/15	18/12	0.51
Birth weight (kg)	3.28 ± 0.32	3.22 ± 0.42	0.54
vWF (ng/ml)	11804.38 (2793.55 – 30280.01)	17727.80 (8267.68 – 40286.38)	**0.001**
ET-1 (pg/ml)	16.47 (0.01 – 236.38)	23.27 (0.16 – 142.20)	0.34
VE-cadherin (ng/ml)	4730.31 ± 1439.13	4120.03 ± 1518.71	0.11

BMI, body mass index; ET-1, endothelin-1; GDM, gestational diabetes mellitus; HbA1c, glycated hemoglobin A1c; vWF, Von Willebrand Factor; VE-cadherin, vascular endothelial-cadherin. Data are expressed as mean ± SD (range) or number (percentage); ^*^as determined by continuity correction; values in bold indicate statistical significance.

**Table 2:** Comparison of sonographic parameters in fetuses from control and GDM groups.

Sonographic parameters	Controls (N = 31)	GDM (N = 30)	*P* value
APV (cm/s)	70.67 (48.33 – 79.00)	40.50 (28.00 – 53.33)	**0.001**
UA-PI	0.82 ± 0.12	0.87 ± 0.14	0.09
LVLW (mm)	3.58 ± 0.30	3.53 ± 0.40	0.53
IVS (mm)	3.70 (3.0 – 3.8)	3.50 (3.0 – 4.9)	0.11
RVFW (mm)	3.70 (3.2 – 4.2)	3.50 (3.0 – 4.5)	0.07

APV, aortic propagation velocity; UA-PI, umbilical artery–pulsatility index; LVLW, left ventricular lateral wall; IVS, interventricular septum; RVFW, right ventricular free wall.

Values in bold indicate statistical significance.

**Table 3:** Correlation between ultrasonic measurements with vWF as well as HbA1c and glucose.

Variables	APV	vWF	HbA1c	Fasting blood glucose	1-h plasma glucose	2-h plasma glucose
vWF	−0.58 (**<0.001**)	1 (1)	0.26 (**0.04**)	0.33 (**0.01**)	0.35 (**0.01**)	0.29 (**0.02**)
APV	1 (1)	−0.58 (**<0.001**)	−0.46 (**<0.001**)	−0.59 (**<0.001**)	−0.62 (**<0.001**)	−0.63 (**<0.001**)
UA-PI	−0.13 (0.30)	0.01 (0.96)	0.1 (0.46)	0.01 (0.93)	0.04 (0.78)	0.08 (0.53)
LVLW	0.07 (0.58)	−0.24 (0.07)	0.02 (0.86)	−0.02 (0.86)	−0.04 (0.75)	0.07 (0.59)
IVS	0.20 (0.13)	−0.19 (0.14)	−0.09 (0.51)	−0.14 (0.30)	−0.14 (0.3)	−0.05 (0.71)
RVFW	0.19 (0.14)	−0.16 (0.21)	−0.15 (0.25)	−0.16 (0.22)	−0.19 (0.13)	−0.13 (0.32)

APV, aortic propagation velocity; UA-PI, umbilical artery–pulsatility index; LVLW, left ventricular lateral wall; IVS, interventricular septum; RVFW, right ventricular free wall; vWF, Von Willebrand Factor.

Data are expressed as correlation coefficient (P value); values in bold indicate statistical significance.

Considering that other factors such as ventricular wall thickness and placental resistance may potentially have impacts on the propagation of aortic flow, we further analyzed the relationships between APV and ventricular wall thickness and UA-PI. However, we did not detect any associations between APV and ventricular wall thickness or UA-PI. There were also no associations between vWF and ventricular thickness or UA-PI (**Table 3**).

## Discussion

In this study, we found that APV measured by fetal echocardiography was decreased in fetuses from women with GDM and that it correlated with serum levels of vWF in umbilical cord blood. These results indicate that measurement of fetal APV may represent a promising non-invasive and simple method to assess endothelial function in fetuses. Although APV has been used and validated in adults with vascular disease and heart disease (14, 16), it has not been previously studied in GDM and has not been examined in the fetus. To the best of our knowledge, our study is the first to assess the relationship between fetal APV measurements and endothelial dysfunction, reflected by an associated biomarker in patients with GDM.

Maternal glucose can be delivered to fetuses *via* the fetoplacental circulation, which would cause fetal hyperglycemia and reactive hyperinsulinemia. Therefore, this may cause endothelial dysfunction within the fetal micro- and macrocirculation or even metabolic factors that may increase risk of CVD in offspring (20, 21). Previous studies showed that GDM is associated with increased oxidative stress and overexpression of inflammatory cytokines, both of which might lead to endothelial dysfunction and future vascular disease (8, 11). Endothelial cells that are stimulated or damaged show changes in a variety of biomarkers such as vWF, ET-1 and VE-cadherin, which are markers of endothelial cell injury (22). Previous studies reported the presence of endothelial dysfunction in GDM fetuses as indicated by alterations of endothelial markers in cord blood (11). In addition, 6–13 years old children born from mothers with GDM have a worse cardiovascular risk profile and increased adhesion molecules compared with controls, suggesting persistent damaging effects of GDM *in utero* on endothelial function (23).

VE-cadherin, a vascular endothelial cell specific cadherin, has been used to indirectly evaluate elevated vascular permeability and assess the degree of endothelial dysfunction (22). ET-1, as a potent endothelial-derived constrictor of vascular smooth muscle, may lead to endothelial dysfunction. However, in this study, we did not observe any significant difference in ET-1 and VE-cadherin levels between the GDM and control groups, which could be related to good glycemic control in this study. Endothelial dysfunction is defined by an injured vascular reactivity with a pro-inflammatory state, but it also refers to a prothrombotic state. vWF, a glycosylated protein, is primarily synthesized in endothelial cells and circulates into the bloodstream to promote platelet adhesion and aggregation (24). Several studies have indicated that elevated expression of vWF in plasma suggests endothelial injury and endothelial dysfunction (22, 24). Although whether the increased plasma concentration of vWF is a cause or consequence of endothelial dysfunction remains unclear, one study showed that reduced vWF overexpression can protect endothelial cells from harmful stimulation from high Angiotensin II (25). To date, there is no noninvasive method for prenatal evaluation of fetal vascular endothelial function. Moreover, FMD, which is used to evaluate endothelial dysfunction in adults, is challenging *in utero* because it requires the use of a blood pressure cuff (13). Previous studies showed that color M-mode APV may serve as a potential marker of endothelial dysfunction in the origin of descending aorta in adults (15–17).

After stimulation, stress or damage to endothelial cells, the resultant vascular endothelial impairment increases vascular fibrosis of wide arteries, leading to increased downstream resistance through the thickening and stiffening of the arterial wall. A decrease in the flow propagation speed within the arterial lumen can occur, which corresponds to the decreased APV values. Gunes et al. demonstrated that APV is associated with carotid and coronary atherosclerosis and brachial endothelial function in adults (17). Furthermore, Ari et al. reported that APV was not correlated with aortic stiffness in healthy individuals (26). However, the authors used aortic distensibility and aortic strain to evaluate aortic stiffness rather than a validated aortic stiffness evaluation method such as aortic pulse wave velocity or an endothelial functional metric such as FMD. In this study, the fetal APV values were decreased in the GDM cohort compared with controls, suggesting a different fetal vascular functional state in the two groups. These apparently discrepant findings may be explained by pathologic conditions, such as metabolic diseases or CVDs, which may have a composite effect on aortic elasticity indices and flow propagation.

Interestingly, APV at late gestation correlated well with serum levels of the endothelial functional marker vWF in fetal cord blood. These findings were consistent with those obtained in adults (16, 17), suggesting that APV measurement can serve as a novel, non-invasive method for estimation of fetal vascular function. These results also suggest that APV may help in risk stratification and help improve management strategies such as diabetic control to obtain long-term benefits for patients. However, the correlation between APV and endothelial function in this study was moderate. The moderate correlation may be from the small sample of patients or the well-controlled diabetes in this study. Thus, further investigation with a larger study population is needed to better understand the role of APV in the recognition of fetal endothelial dysfunction and its effects on short-term and long-term risks of cardiometabolic diseases in the offspring of women with GDM.

There were other potential factors including the placental function and cardiac hypertrophy that may affect the flow propagation speed. The placenta from patients with GDM is known to be abnormal, as evidenced by the increase in placental vascular resistance such as elevated UA-PI (27). Extreme conditions such as poorly controlled diabetes may exceed the placental homeostatic capacity, with potentially adverse outcomes for the fetus (28). However, Leung et al. suggested that Doppler indices of the placenta were not associated with maternal diabetes mellitus (29), which was in line with our findings. Furthermore, there was no evident hypertrophy in this study, which may be explained by the well-controlled diabetes in the current study.

The measurement of fetal APV has several advantages. APV measurement is completed within a few minutes and does not require invasive tools. It does not use cuff inflation or deflation in the patient’s arm like FMD and it does not require a particular fetal position. Operator skill is not critical since the assessment is performed in the sagittal view of aortic arch, which is a routine obstetrical screening scan. Fetal APV measurements in our study showed with good intra- and interobserver agreement. These findings indicate measurement of APV as a simple and practical method for the assessment of vascular function.

Prenatal noninvasive detection of endothelial function is valuable in many situations such as GDM, preeclampsia and maternal hypercholesterolemia. In this study, we demonstrated that APV can help identify initial alterations of vascular function, even in patients with well-controlled GDM. APV may be used in fetuses referred for a fetal echocardiography, to improve cardiovascular risk estimation, for better selection of high-risk individuals, and for corresponding preventive interventions.

### Limitations

This study had several limitations. We did not use more validated biomarkers of endothelial dysfunction to better determine the role of APV. In addition, we were unable to conduct follow-up of neonatal endothelial function because of the COVID-19 global pandemic. More study is necessary to investigate whether endothelial dysfunction is dynamically altered throughout gestation to the neonatal period or even childhood. This was also a single-center prospective study with a small patient group. Thus, a large, multicenter validation study is warranted to identify the role of APV measurements.

## Conclusion

Intrauterine hyperglycemia may be associated with impaired endothelial function assessed by APV by fetal echocardiography and vWF levels a biomarker of endothelial function. Color M-mode propagation velocity of aorta is a non-invasive, practical method and correlates well with GDM and endothelial function. This novel metric may be helpful in the recognition of initial early vascular functional alterations.

## Data Availability Statement

The datasets generated for this study are available on request to the corresponding authors.

## Ethics Statement

The studies involving human participants were reviewed and approved by the ethics committee of the Guangzhou Women and Children’s Medical Center. The patients/participants provided their written informed consent to participate in this study. Written informed consent was obtained from the individual(s) for the publication of any potentially identifiable images or data included in this article.

## Author Contributions

HongYW, WST, and YC conceived, designed and supervised the study. HongYW, WST and YC drafted and revised the manuscript. HongYW and DH performed the fetal echocardiography. FZ, GT, and YJ provided nutritional counseling and care for patients. JL, WL, and HaiYW analyzed and interpreted the data. WST and YC provided writing assistance and review of the manuscript in English. All authors of this manuscript approved that HongYW and WST shared correspondence authorship for the work. All authors contributed to the article and approved the submitted version.

## Funding

This research was supported by the Funding of Guangzhou Institute of Pediatrics/Guangzhou Women and Children’s Medical Center (grant number: IP-2018-003).

## Conflict of Interest

The authors declare that the research was conducted in the absence of any commercial or financial relationships that could be construed as a potential conflict of interest.

## Publisher’s Note

All claims expressed in this article are solely those of the authors and do not necessarily represent those of their affiliated organizations, or those of the publisher, the editors and the reviewers. Any product that may be evaluated in this article, or claim that may be made by its manufacturer, is not guaranteed or endorsed by the publisher.

## Abbreviations

APV, aortic propagation velocity; FMD, flow-mediated vasodilation; IVS, interventricular septum; LVLW, left ventricular lateral wall; RVFW, right ventricular free wall; UAPI, umbilical artery–pulsatility index.
